# Abdominal aortic aneurysms ruptured to the vena cava: a case series and literature review

**DOI:** 10.1590/1677-5449.200174

**Published:** 2021-04-05

**Authors:** Carolina de Oliveira, Bruno Pagnin Schmid, Giovani José Dal Poggetto Molinari, Ana Terezinha Guillaumon

**Affiliations:** 1 Universidade Estadual de Campinas – UNICAMP, Campinas, SP, Brasil.

**Keywords:** aneurysm, ruptured, aortic aneurysm, abdominal, arteriovenous fistula, case series, vascular fistula, ruptura aórtica, aneurisma aórtico, fístula vascular, relatos de casos, fístula arteriovenosa

## Abstract

Ruptured abdominal aortic aneurysms (RAAA) evolving aortocaval fistula (AF) can have catastrophic hemodynamic effects. Surgical repair is imperative, but the optimal technical approach is still under debate. Our objective is to present 3 cases treated with endovascular repair (EVAR) at a University Hospital. Case #1, a 71-year-old man presenting a 7.1cm RAAA with AF, repaired with a monoiliac stent graft and femoral-femoral bypass; Case #2, a 76-year-old man presenting a 9.9cm RAAA with AF, repaired with a bifurcated stent graft; Case #3, a 67-year-old man with previous history of EVAR, presenting a type 3 endoleak with late rupture related to AF, repaired with a tubular stent graft. All cases unfolded with delayed recovery and significant complication rates, although AF symptoms had resolved by hospital discharge. EVAR techniques for AF may require secondary interventions but are feasible, despite the lack of consensus, considering the rarity of this RAAA presentation.

## Resumo

Aneurismas de aorta abdominal rotos (AAAR) com evolução para fístula aorto-cava (FAC) podem apresentar consequências hemodinâmicas catastróficas. A correção cirúrgica é mandatória, embora não haja consenso sobre a técnica operatória. Apresentamos uma série de três casos operados em hospital universitário pela técnica endovascular. No primeiro caso, um homem de 71 anos apresentou AAAR de 7,1 cm com FAC, submetido a correção por endoprótese monoilíaca e enxerto femoral cruzado. No segundo, um homem de 76 anos apresentou AAAR de 9,9 cm com FAC submetido a colocação de endoprótese bifurcada. O terceiro caso era de um homem de 67 anos com histórico de EVAR e *endoleak* tipo 3, com ruptura tardia para veia cava, tratado com extensão aórtica. Todos apresentaram evolução pós-operatória prolongada com significativas complicações, entretanto com boa resolução dos sintomas à alta hospitalar. A EVAR é uma técnica promissora para o tratamento de FAC, embora com taxa de reintervenção significativa.

## INTRODUCTION

Aortocaval fistula (AF) is defined as anomalous communication between the aorta and the inferior vena cava.^1^ Abdominal aortic aneurysm ruptured to the vena cava is a rare finding, with a reported incidence of less than 1% in ruptured abdominal aortic aneurysms (RAAA), but can lead to catastrophic hemodynamic effects with acute high-output heart failure.^2^^,^ 3 Just a few cases have been reported in the literature and there is limited experience even at large centers. There is thus a lack of consensus on how to manage this disease. Surgical repair is imperative, but the optimal technical approach, whether open or endovascular repair, is still under debate.^4^ Our objective is to present a series of 3 cases that were treated with endovascular techniques at a University Hospital and discuss clinical presentations of the disease and the technical operative options.

## CASE DESCRIPTIONS

This is a retrospective, single-center series of 3 consecutive cases (3 men) treated with EVAR from April 2017 to November 2017 at a University Hospital in Brazil. The patients’ characteristics, according to the Society of Vascular Surgery reporting standards guidelines, clinical presentations, and imaging findings are shown in Table 1.^5^ All patients underwent angiotomography at admission in the hospital’s emergency department.

**Table 1:** Patients’ characteristics, clinical presentations, and imaging findings.

Case	1	2	3
Date of operation	May 9, 2017	December 11, 2017	April 26, 2017
Age (years)	71	76	67
Gender	Male	Male	Male
Hypertension	No	Yes (requiring >2 drugs)	Yes (requiring >2 drugs)
Tobacco use	Yes (quit 20 years ago)	No	Yes (quit 3 years ago)
Diabetes	No	No	No
Renal status	Normal	Evidence of renal disease, GFR> 90	Normal
Cardiac status	Asymptomatic, with normal electrocardiogram	Poorly compensated, congestive heart failure	Asymptomatic, with normal electrocardiogram
Previous surgical procedures	No	No	EVAR AFX (Endologix® Inc; Irvine, CA) for RAAA
Clinical presentation	Abdominal pain, central pulsatile mass, loud continuous machinery-like midabdominal bruit	Abdominal pain, central pulsatile mass, acute renal failure, hematuria	Abdominal pain, central pulsatile mass, bilateral limb and scrotal edema, hematuria
Hemodynamic presentation	Stable	Stable	Stable
Imaging findings	RAAA (7.6cm, infrarenal) with AF	RAAA (9.9 cm, infrarenal) with AF	Type 3 Endoleak with late rupture related to AF (and possible left type 1b endoleak)

RAAA = Ruptured abdominal aortic aneurysm; AF = aortocaval fistula; EVAR = endovascular aneurysm repair; GFR = glomerular filtration rate (mL/min/1.73 m^2^).

We used the OsiriX MD Imaging Software® (Pixmeo Labs; Geneva, Switzerland) for preoperative EVAR planning in all cases. All surgical procedures were performed through an open bilateral common femoral access with general anesthesia and the patient in a supine position. All procedures were headed by an experienced vascular surgeon assisted by 2 vascular surgery residents and 1 vascular surgery fellow. Post-operatively, patients were instructed to take antiplatelet medication and statins (100mg/day of acetylsalicylic acid and 40mg/day of Simvastatin®) after hospital discharge. Detailed surgical procedures and patients’ outcomes are presented in Table 2.

**Table 2:** Details of surgical procedures and patient outcomes.

	Case 1	Case 2	Case 3
Surgical procedure	EVAR Endurant II, (Medtronic® Vascular Inc., Santa Rosa, CA) Monoiliac stent graft + proximal extension + iliac extension and femoral-femoral bypass	EVAR Zenith Flex, (Cook Medical® Inc., Bloomington, IN) Bifurcated stent graft	EVAR Endurant II, (Medtronic® Vascular Inc., Santa Rosa, CA) Tubular aortic stent graft + LIIA coil embolization +external left iliac extension
Intraoperative red blood cell transfusion (#packs)	5	6	3
Total operating time (hours)	5.5	4	4 (first operation) 5 (reintervention)
Complications	Dacron infection	Type 2a Endoleak, Acute renal failure (needed temporary dialysis), *Klebsiella ESBL* bacteremia, Pulmonary thromboembolism	Acute renal failure (needed temporary dialysis)
Reintervention	Femoral graft excision and autologous great saphenous vein femoral-femoral bypass (postoperative day 41).	No	EVAR Endurant II, Medtronic ® Vascular Inc., Santa Rosa, CA Monoiliac stent graft + right iliac extension + LCIA occlusion + femoral-femoral bypass (postoperative day 14).
Length of hospital stay(days)	25	81	34
Imaging follow-up (Angiotomography)	2.7 years	23 days	1.5 years
Clinical follow-up (years)	3.07	Death - 4 months after surgical repair (acute myocardial infarction)	2.97

ESBL = extended-spectrum β-lactamases; LIIA = left internal iliac artery; LCIA = left common iliac artery.

**Figure 1:**
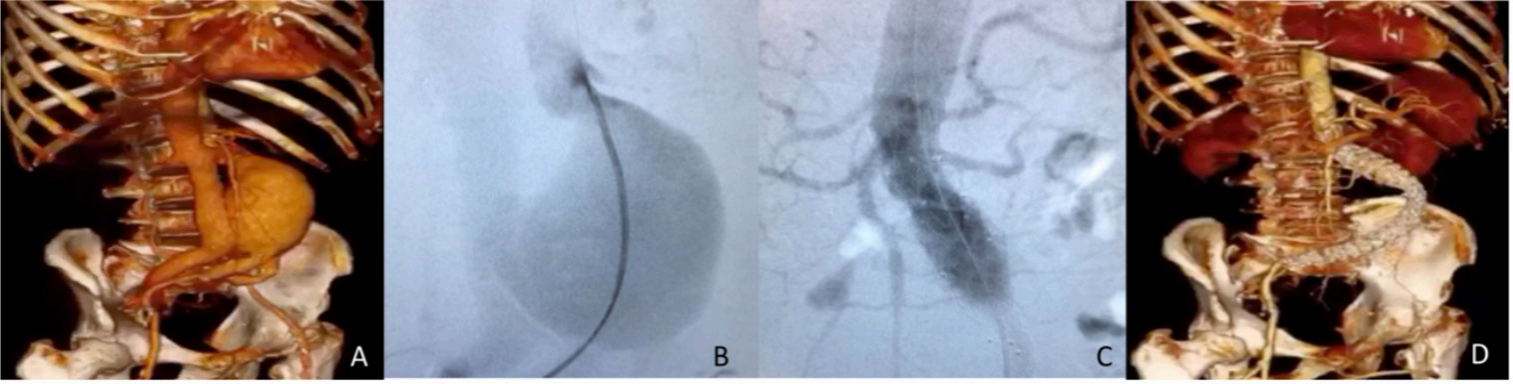
Case#1 (A) Preoperative 3D-CTA showing the RAAA involving an AF, demonstrated by early filling of the inferior vena cava; (B) Intraoperative DSA; (C) Intraoperative DSA after endograft deployment; (D) Postoperative 3D-CTA showing complete AF resolution. AF = Aortocaval fistula; DSA = digital subtraction angiography; RAAA = ruptured abdominal aortic aneurysms; 3D-CTA = three-dimensional-by-volume CT angiography.

Figures 1, [Fig gf02] to 3 illustrate the preoperative, intraoperative, and postoperative imaging exams from each case.

**Figure 2 gf02:**
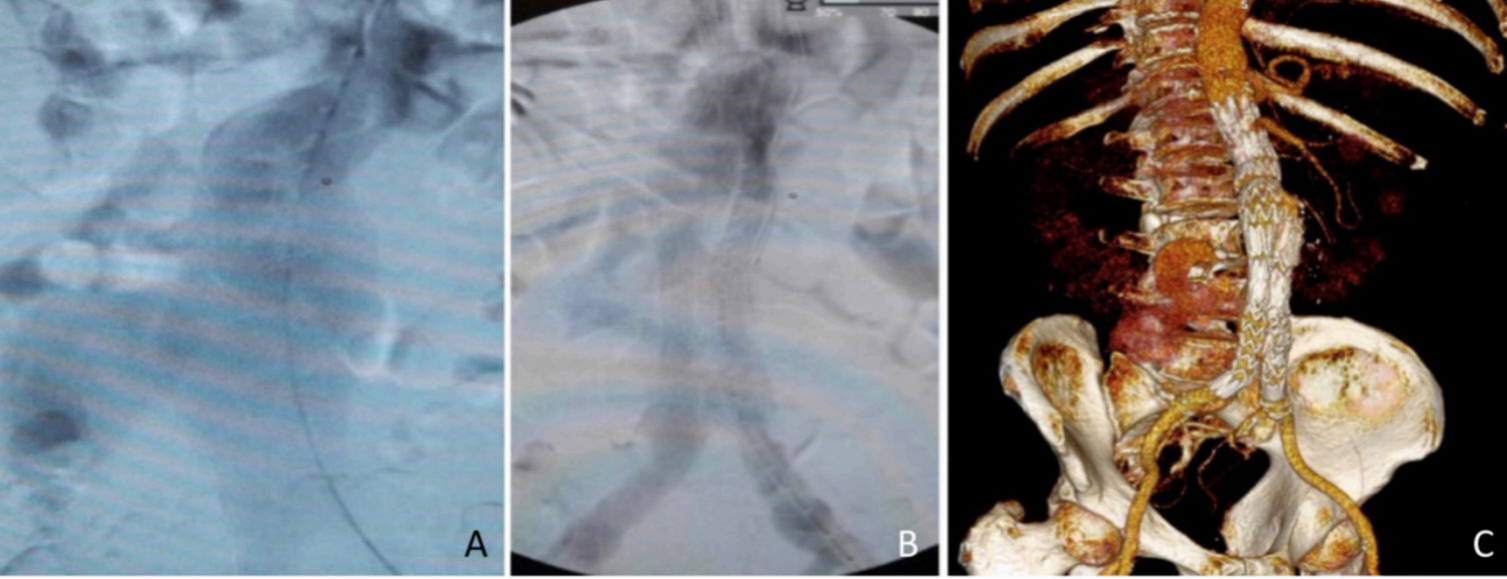
Case#2 (A) Intraoperative DSA showing the RAAA involving an AF, demonstrated by early filling of the inferior vena cava; (B) Intraoperative DSA after endograft deployment; (C) Postoperative 3D-CTA showing complete AF resolution and adequate sealing. AF = Aortocaval fistula; DSA = digital subtraction angiography; RAAA = ruptured abdominal aortic aneurysms; 3D-CTA = three-dimensional-by-volume CT angiography.

**Figure 3:**
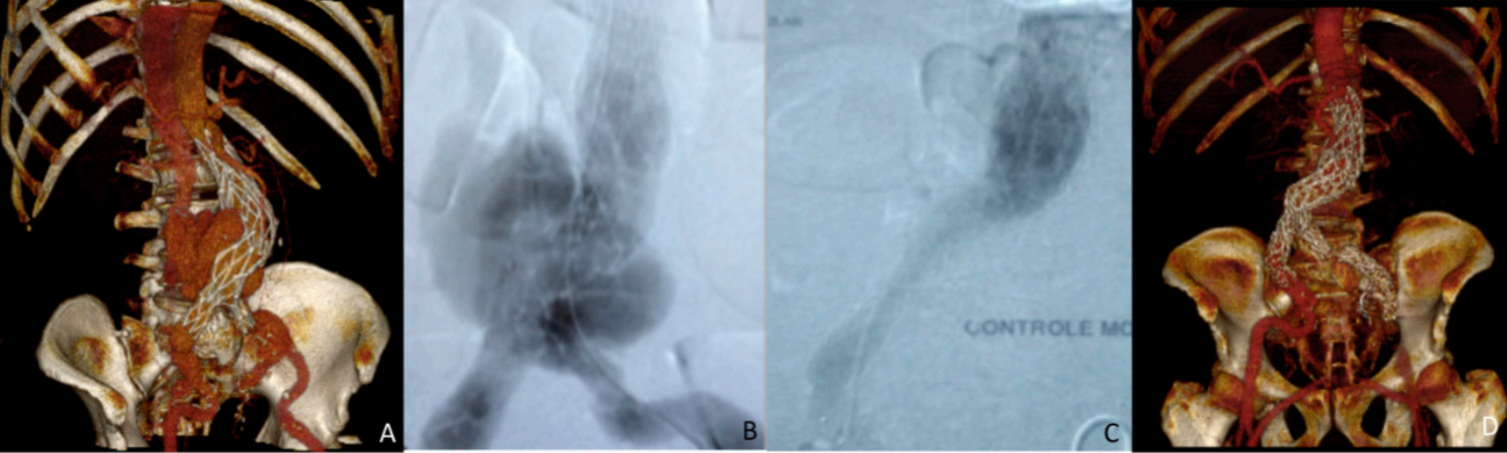
Case #3 (A) Preoperative 3D-CTA showing type 3 endoleak with late rupture related to an AF; (B) Intraoperative DSA showing the type 3 endoleak and AF with filling of the inferior vena cava; (C) Intraoperative DSA demonstrating apparent resolution after endograft deployment; (D) Postoperative 3D-CTA showing complete AF resolution and adequate sealing. AF = Aortocaval fistula; DSA = digital subtraction angiography; 3D-CTA = three-dimensional-by-volume CT angiography.

## DISCUSSION

Syme is credited with being the first to describe an aortocaval fistula in 1831.^6^ The first attempt to repair AF surgically was reported in 1938 by Lehman, but the patient did not survive the initial aortic dissection.^7^ Over 17 years later, Dr. Cooley reported the first successful AF repair using an aorto biiliac bypass.^8^ In 1998, Beveridge et al. reported the first endovascular repair of AF using a bifurcated stent-graft and right internal iliac artery coil embolization.^9^

The clinical presentation of an AAA with AF usually involves a pulsatile abdominal mass, abdominal pain, and an abdominal machinery-like bruit.^10^ Other possible symptoms include dyspnea, lower-extremity edema and ascites due to venous hypertension, and acute renal failure consequent to decreased median arterial pressure.^10^ The pathophysiological mechanism responsible for these systemic effects attributable to AF is the drop in total peripheral resistance. Compensatory mechanisms include enhanced venous return, cardiac contractility, and increased heart rate and vascular resistance secondary to direct sympathetic effects and to enhanced secretion of renin from the kidney. The high output heart failure contributes to this condition’s high mortality.^3^ Pulmonary embolism is another possible serious complication that can result from emboli originating within the aneurysm sac trespassing on the IVC.^10^ Contrast-enhanced multislice angiotomography confirms the diagnosis.

Considering these severe clinical outcomes, surgical repair is imperative. However, the optimal technical approach is still under debate. Open repair has been the recommended therapy for years, but is associated with significant morbidity, mortality, costs, and delayed recovery.^11^ Endovascular procedures for non-complicated RAAA became popular due to lower morbidity and mortality and rapid hospital discharge. Thus, some authors present promising results with this specific technique for management of AF.^11^^-^ 13

In 2016, Orion et. al. published a literature review of 40 articles with a total of 67 treated patients (26 in the endovascular - EVAR group and 41 in the open repair - OR group).^12^ Five deaths (19%) occurred in the EVAR group and five (12%) occurred in the OR group (p=0.910). Postoperative complications occurred in 13 patients (50%) in the EVAR group and in 15 patients (36%) in the OR group (p=0.327). Therefore, EVAR presents theoretical benefit, but was not associated with reductions in complications or deaths in this study.^12^

The endovascular approach has proved to be a valuable technique in management of this challenging and life-threatening condition, with mortality rates in the literature that range from 16% to 66%, especially in patients with poor cardiac status who wouldn’t be suitable for OR.^14^

All three patients survived the emergency treatment, were discharged hospital, and had resolution of symptoms, although one patient needed vascular reintervention to a treat femoral graft infection and one patient to ensure optimal AF sealing. The choice between open or endovascular repair was made based upon clinical and hemodynamic status, anatomical characteristics, and prompt availability of devices for endovascular treatment. Nevertheless, we believe it is important that the surgeon is familiar with both techniques to provide optimal acute care.

Hopefully, this case series can contribute to understanding of the disease and its clinical outcomes, presenting a selection of different surgical strategies used, and stimulate further discussion and research.

This study was approved by the Institutional Ethics Committee (Decision number 2.886.117).
